# Supplementary material for: Role and mechanism of NCAPD3 in promoting malignant behaviors in gastric cancer
Source: Front Pharmacol. 2024 Apr 22;15:1341039. doi: 10.3389/fphar.2024.1341039 (PMC11070777; doi:10.3389/fphar.2024.1341039)
Supplement: Supplementary file 11 [file DataSheet2.ZIP › GSEA/immunologic/my_analysis.Gsea.1599462003817/GSE17974_CTRL_VS_ACT_IL4_AND_ANTI_IL12_6H_CD4_TCELL_UP.html]

Details for gene set GSE17974\_CTRL\_VS\_ACT\_IL4\_AND\_ANTI\_IL12\_6H\_CD4\_TCELL\_UP[GSEA]

|  || Dataset | filtered\_dataset.sample\_info.cls#WT\_versus\_NCAPD3\_MUT |
| Phenotype | sample\_info.cls#WT\_versus\_NCAPD3\_MUT |
| Upregulated in class | NCAPD3\_MUT |
| GeneSet | GSE17974\_CTRL\_VS\_ACT\_IL4\_AND\_ANTI\_IL12\_6H\_CD4\_TCELL\_UP |
| Enrichment Score (ES) | -0.54444516 |
| Normalized Enrichment Score (NES) | -2.6519477 |
| Nominal p-value | 0.0 |
| FDR q-value | 0.003412596 |
| FWER p-Value | 0.013 |
Table: GSEA Results Summary

  

Fig 1: Enrichment plot: GSE17974\_CTRL\_VS\_ACT\_IL4\_AND\_ANTI\_IL12\_6H\_CD4\_TCELL\_UP      
 Profile of the Running ES Score & Positions of GeneSet Members on the Rank Ordered List

  

| SYMBOL | TITLE | RANK IN GENE LIST | RANK METRIC SCORE | RUNNING ES | CORE ENRICHMENT || 1 | 7109 | TRAPPC10 | 475 | 0.488 | -0.3056 | No |
| 2 | 130502 | TTC32 | 517 | 0.465 | -0.2999 | No |
| 3 | 360 | AQP3 | 857 | -0.263 | -0.5245 | Yes |
| 4 | 6515 | SLC2A3 | 873 | -0.283 | -0.5138 | Yes |
| 5 | 8848 | TSC22D1 | 899 | -0.324 | -0.5073 | Yes |
| 6 | 4616 | GADD45B | 904 | -0.330 | -0.4850 | Yes |
| 7 | 4209 | MEF2D | 910 | -0.337 | -0.4630 | Yes |
| 8 | 9414 | TJP2 | 916 | -0.345 | -0.4404 | Yes |
| 9 | 9314 | KLF4 | 972 | -0.389 | -0.4506 | Yes |
| 10 | 51646 | YPEL5 | 1014 | -0.413 | -0.4487 | Yes |
| 11 | 23764 | MAFF | 1039 | -0.436 | -0.4329 | Yes |
| 12 | 80714 | PBX4 | 1054 | -0.449 | -0.4089 | Yes |
| 13 | 3669 | ISG20 | 1097 | -0.475 | -0.4031 | Yes |
| 14 | 54541 | DDIT4 | 1133 | -0.502 | -0.3903 | Yes |
| 15 | 4084 | MXD1 | 1167 | -0.535 | -0.3734 | Yes |
| 16 | 7128 | TNFAIP3 | 1198 | -0.573 | -0.3515 | Yes |
| 17 | 2043 | EPHA4 | 1228 | -0.602 | -0.3267 | Yes |
| 18 | 54491 | FAM105A | 1241 | -0.619 | -0.2883 | Yes |
| 19 | 26137 | ZBTB20 | 1244 | -0.622 | -0.2425 | Yes |
| 20 | 80201 | HKDC1 | 1289 | -0.681 | -0.2225 | Yes |
| 21 | 57561 | ARRDC3 | 1324 | -0.744 | -0.1905 | Yes |
| 22 | 5997 | RGS2 | 1336 | -0.770 | -0.1399 | Yes |
| 23 | 84959 | UBASH3B | 1338 | -0.776 | -0.0817 | Yes |
| 24 | 54463 | FAM134B | 1356 | -0.815 | -0.0320 | Yes |
| 25 | 10397 | NDRG1 | 1380 | -0.925 | 0.0216 | Yes |
Table: GSEA details [plain text format]

  

Fig 2: GSE17974\_CTRL\_VS\_ACT\_IL4\_AND\_ANTI\_IL12\_6H\_CD4\_TCELL\_UP      
 Blue-Pink O' Gram in the Space of the Analyzed GeneSet

  

Fig 3: GSE17974\_CTRL\_VS\_ACT\_IL4\_AND\_ANTI\_IL12\_6H\_CD4\_TCELL\_UP: Random ES distribution      
 Gene set null distribution of ES for **GSE17974\_CTRL\_VS\_ACT\_IL4\_AND\_ANTI\_IL12\_6H\_CD4\_TCELL\_UP**
